# Topological and geometric analysis of cell states in single-cell transcriptomic data

**DOI:** 10.1093/bib/bbae176

**Published:** 2024-04-18

**Authors:** Tram Huynh, Zixuan Cang

**Affiliations:** Department of Mathematics and Center for Research in Scientific Computation, North Carolina State University, NC 27695, USA; Department of Mathematics and Center for Research in Scientific Computation, North Carolina State University, NC 27695, USA

**Keywords:** scRNA-seq, cell state, transition cell, curvature, persistent homology

## Abstract

Single-cell RNA sequencing (scRNA-seq) enables dissecting cellular heterogeneity in tissues, resulting in numerous biological discoveries. Various computational methods have been devised to delineate cell types by clustering scRNA-seq data, where clusters are often annotated using prior knowledge of marker genes. In addition to identifying pure cell types, several methods have been developed to identify cells undergoing state transitions, which often rely on prior clustering results. The present computational approaches predominantly investigate the local and first-order structures of scRNA-seq data using graph representations, while scRNA-seq data frequently display complex high-dimensional structures. Here, we introduce scGeom, a tool that exploits the multiscale and multidimensional structures in scRNA-seq data by analyzing the geometry and topology through curvature and persistent homology of both cell and gene networks. We demonstrate the utility of these structural features to reflect biological properties and functions in several applications, where we show that curvatures and topological signatures of cell and gene networks can help indicate transition cells and the differentiation potential of cells. We also illustrate that structural characteristics can improve the classification of cell types.

## INTRODUCTION

Single-cell RNA sequencing (scRNA-seq) provides a high-throughput method to measure gene expression profiles of individual cells, which enables the dissecting of cellular heterogeneity at an unprecedented resolution [1]. Many computational methods have been developed for scRNA-seq data and have revealed various new cell types and differentiation trajectories [2]. In scRNA-seq analysis, clustering and trajectory inference are two main analysis tasks. They are often performed in a reduced-dimensional space where a metric describes cell similarity. In clustering, each cell group potentially represents a cell type, often annotated by confirming marker genes with prior knowledge. In trajectory inference, a graph is often constructed by connecting cells with similar gene expression profiles upon which minimal spanning trees or graph coarsening can be performed to summarize the trajectory structures. Identifying transition cells between states is crucial to inferring local transitions between stable cell states. Compared to cells clearly belonging to a cell type, transition cells between cell types are often not captured as effectively in scRNA-seq data because of the instability of transition states. Furthermore, the biological properties of cell groups, such as differentiation potential, are mainly annotated using prior knowledge [3]. Predicting differentiation potential can annotate global temporal directionality in a dataset. Computational methods for exploring transition states and unsupervised analysis of differentiation potential are still underexplored.

Recently, several methods have been developed to study the transition states between cell types. QuanTC [4] and scRCMF [5] perform nonnegative matrix factorizations on the cell-by-cell similarity matrix and cell-by-gene expression matrix, respectively, with each factor representing a cell type. The entropy of the assignment scores of each cell to the cell types is used as an indicator of the transition cells. Soft clustering algorithms can also derive soft assignment scores to determine pure and transition cells such as SOUP [6], DBCTI [7] and scTite [8], using predefined criteria or entropy. MuTrans [9] models the scRNA-seq data as a dynamical system based on a cell fate dynamical manifold determined from clustering and identifies transition cells also using the entropy of the assignment scores of cells to sinks. Cabybara [10] utilizes the vast reference databases of annotated bulk and single-cell transcriptomic data to assign cell types to single cells and identifies cells predicted to have hybrid cell types as transition cells. These methods are based on data clustering, which often requires certain clustering parameters such as number of clusters and clustering resolution, or classification of data using reference training data. Here, we aim to explore the structures underlying single-cell data to infer transition cells without relying on clustering or classification results.

Analyzing pluripotency or differentiation potential of cell groups is valuable for refining structures and assigning global directions to the pseudo-temporal trajectories inferred from scRNA-seq data. With the accumulation of large-scale networks such as gene regulatory and protein–protein interaction networks, and computational methods to infer large-scale gene networks from scRNA-seq data such as correlation-based ones [11, 12], there exists an opportunity to infer pluripotency by examining the structures of gene networks. For example, entropy [13, 14] and curvature [15] in gene networks have been used to reflect cell pluripotency. These methods use global summaries of the local properties of gene networks. Here, we aim to further use topological methods for multiscale analysis of both local and global structures of gene networks.

High-dimensional scRNA-seq data assemble a complex heterogeneous manifold, while the emerging field of topological and geometric data analysis (TGDA) [16] specifically aims to systematically extract structural information from such complex structures. Mapper [17] is one of the main tools in TGDA that derives a structural abstraction of often high-dimensional data and has been applied to scRNA-seq data to extract a simplified manifold underlying the data [18]. Another main tool, persistent homology [19–21], systematically examines topological characteristics of different dimensions and at various geometric scales. Persistent homology has found its applications in various biological fields such as analyzing neural activity data [22] and structure-based biomolecular property predictions [23, 24]. Persistent homology is generally applicable for different types of data including point clouds, volumetric data [25], and networks [26]. Its application in scRNA-seq data, however, remains underexplored.

Here, our objective is to explore the usage of TGDA tools, specifically graph curvature [27] and persistent homology [19, 20], to establish structure-function relationships in scRNA-seq to predict cell properties from the underlying structures of the data. We focus on two types of structures, a network of cells with cells connected based on their gene expression similarities and gene networks associated with each cell. Based on the cell network, we use the Ollivier–Ricci curvature (ORC) [27], a discretization of the Ricci curvature on graphs, local persistent homology [24], and relative persistent homology [28] to identify transition cells, independent of clustering or classification of cell types. For gene networks, we use vertex-based clique complex and edge-weighted Vietoris-Rips complex-based persistent homology to characterize node-weighted knowledge-based gene networks and edge-weighted cell-specific gene networks, respectively. Topological summaries are then related to the pluripotency or differentiation potential of cells. In a more general case, we also explore the usage of topological summaries as additional features in the task of cell-type classification. These utilities are demonstrated in several real datasets with ground truth from scRNA-seq data on real time points or expert annotations.

## RESULTS

### Method overview

**Figure 1:**
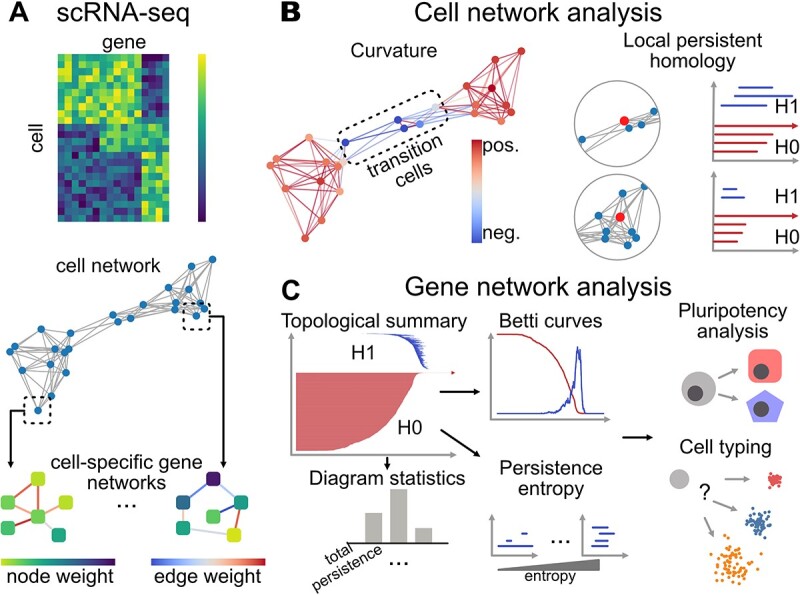
Overview of scGeom. (**A**) The structure of an scRNA-seq data is often represented as cell networks, where the cell-specific gene networks can be constructed for each cell with node weights and edge weights representing gene expression levels and interactions between genes. Here, the cell network is constructed as k-nearest-neighbor graphs of cells, and we adopt two approaches for cell-specific gene networks, a statistical method for constructing edge-weighted cell-specific gene networks, and by assigning node weights as gene expression levels to a prior knowledge-based gene network. (**B**) The local structure of each cell is described by curvatures and local topology, which are related to cell states. The topological features are extracted by persistent homology, where each line segment in the $H_{k}$ ($k$th homology group) persistence barcode represents the scales at which a $k$-dimensional hole appears and disappears. For example, the $H_{0}$ and $H_{1}$ barcodes represent connected components and one-dimensional loops, respectively. (**C**) The structures of cell-specific gene networks are characterized by various topological descriptors that are used to link to cell properties, such as pluripotency and cell types.

To explore the structure-function relationship underlying single-cell data, we develop scGeom, a tool that characterizes the geometric and topological properties of cell networks and gene networks and relates them to the biological properties of cells (Figure 1). The single-cell data are first preprocessed following common pipelines of normalization, highly variable gene selection, and PCA dimension reduction. A cell network denoted by $G_{\mathrm{c}}=(V_{\mathrm{c}},E_{\mathrm{c}})$ is then constructed by building a $k$-nearest neighbor graph with respect to the Euclidean distance of the PCA embeddings (Figure 1A). On $G_{\mathrm{c}}$, a graph curvature is computed for each edge using a discretization of the Ricci curvature on graphs, the ORC [27] that measures the divergence of local geometry from Euclidean space. Specifically, for the edge $e_{ij}\in E_{\mathrm{c}}$, ORC examines the difference between the edge length $e_{ij}$ and the distance between the neighborhoods of $v_{i}$ and $v_{j}$ by optimal transport with the shortest path distance as the ground cost. The curvatures on the nodes are then defined by summing over the corresponding edges. A cell within a community or between communities is likely to have a positive or negative graph curvature, respectively, analogous to the scalar curvature in Riemannian geometry.

The topological structures are characterized by persistent homology [19, 20]. The structure of the network is represented by a growing sequence of simplicial complexes that generalize graphs to higher dimensions. This sequence, called a filtration, captures the structural features at various scales, compared to the use of a fixed graph or simplicial complex. Along the filtration, persistent homology tracks the appearances and disappearances of $k$-dimensional holes and their persistence through the filtration. For example, the $0$-, $1$- and $2$-dimensional holes correspond to connected components, loops and voids. Given a graph or point cloud, persistent homology outputs collections of persistence intervals, also called persistence diagrams, $\mathrm{Dgm}_{k}=[b_{i},d_{i})_{i=1}^{n^{(k)}}$ representing the filtration values corresponding to the appearance ($b_{i}$) and the disappearance ($d_{i}$) of the $k$th homology groups ($H_{k}$) associated with $k$-dimensional holes. The details of the graph curvature and persistent homology are discussed in Sections 3.1 and 3.2. For each cell in the cell network, persistent homology is computed for its local neighborhood. The graph curvature and featurizations of the persistence diagram such as total persistence $\sum _{i}(d_{i}-b_{i})$ and persistence entropy [29–31] are used to distinguish cells in the transition and stable states (Figure 1B). Here, the graph curvatures and topological features are determined solely on the cell network and are independent of clustering of cells. Once transition cells are identified, their interpretations can be derived based on distance or population overlap with other independent clustering of cells.

A cell-specific gene network [11], $G_{\mathrm{g}}$ is constructed to reveal higher-order properties of cells in addition to first-order gene expression levels. Persistence diagrams are computed for $G_{\mathrm{g}}^{i}$ of cell $i$ using filtrations such as edge-weighted Vietoris-Rips complex, resulting in persistence diagrams $\mathrm{Dgm}_{k}(G_{\mathrm{g}}^{i})$. We then turn the persistence diagrams into features, including Betti curves that count the number of homology groups at every filtration value and various statistics of $\mathrm{Dgm}_{k}$ such as total persistence and longest persistence. These features are then used to analyze the pluripotency or differentiation potential of cells and are fed into machine learning methods together with gene expression features to predict cell types (Figure 1C). Details of the methods and data preprocessing can be found in Section 3.

### Identifying transition cells with curvature and local topology

**Figure 2:**
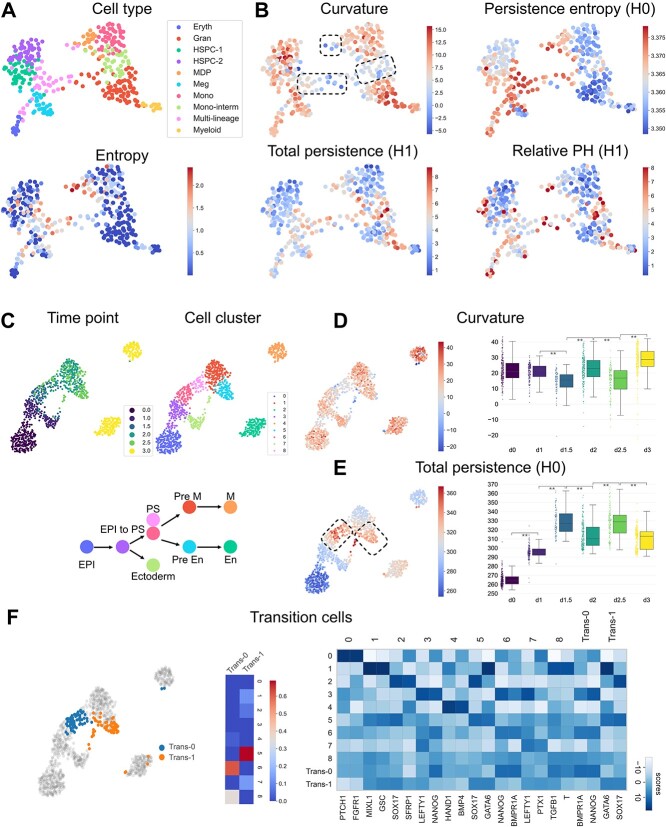
Analysis of transition states. (**A**) A single-cell dataset of myelopoiesis. Applying MuTrans results in the entropy that measures the uncertainty of cluster assignment of a cell that indicates transition cells. (**B**) The structural features in the myelopoiesis dataset computed by scGeom on the cell network for each cell including curvature, local persistent homology, and relative persistent homology. The local persistent homology summarized as persistence entropy and total persistence, and the relative persistent homology described by the Wasserstein distance between the relative persistence diagram and regular persistence diagram are shown. (**C**) A single-cell dataset of iPSC taken at several temporal points from day 0 to day 3 where two transition events occur on day 1.5 and day 2.5. (**D**, **E**) The curvature in the cell network and the total persistence of the local persistent homology output. ** indicates a $P$-value smaller than 1e-10 by Wilcoxon test. (**F**) Transition cells identified from the iPSC dataset, the association of transition groups with predetermined cell groups, and the top two marker genes for each group where groups 0-8 contain cells from the original clustering except the transition cells.

We first analyzed an scRNA-seq data of myelopoiesis that captures several transitional intermediate states during blood cell differentiation [32]. In this dataset, two relatively unstable states, a multi-lineage state and a monocyte intermediate state, were identified by the original study using the ICGS approach [32] and another analysis of the dataset using a physics-based modeling tool MuTrans [9] (Figure 2A). Furthermore, the MuTrans analysis constructed a differentiation landscape and identified transition cells between states depicted by the entropy of the probability score assigned to each state (Figure 2A). Here, based on $k$-nearest-neighbor graphs of cells constructed using PCA embedding, we computed the ORC for each cell. We also computed the topological features of each cell, including local persistent homology on a neighborhood graph centered at the cell and the relative persistent homology of the global structure relative to a small neighborhood of the cell. The local persistent homology captures the multiscale and multidimensional structural characteristics of the local structure centered on each cell, and the resulting persistence diagrams are turned into features by computing the total persistence and the persistence entropy [29]. Relative persistent homology examines the importance of a cell in defining the global structure of the dataset. The resulting persistence diagrams are described by computing the Wasserstein distance between the relative persistence diagram and the regular persistence diagram of the entire dataset. These geometric and topological features were able to highlight both the relatively unstable cell states and the transition cells between states (Figure 2B).

We then analyzed a single-cell dataset of induced pluripotent stem cells (iPSCs) taken at several real time points [33] (Figure 2C). This dataset depicts two major transition events, epiblast (EPI) to primitive-streak (PS) cells and PS to mesenchymal (M) and endodermal (En) cells. Using biological knowledge, the original study [33] projected these two transition events to occur around day 1.5 and day 2.5, respectively. Here, we perform an unbiased analysis without using any prior knowledge. We computed the graph curvature and local persistent homology on the PCA embedding and found a significant decrease in curvature and an increase in total persistence at days 1.5 and day 2.5. A smaller curvature indicates bridges between stable states (Figure 2D). For topology, more significant topological characteristics, for example, higher total persistence, reflect divergence from trivial structures and thus reveal the transition processes (Figure 2E). Using the $H_{0}$ total persistence, we identified transition cells that were separated into two classes by clustering (Figure 2F). The association of the two transition groups with the original clusters is determined by the overlapping population between each transition group and the original cluster. Then, a modified clustering of data was created by introducing the two new transition clusters and removing the transition cells from the original clusters. Marker gene analysis based on this modified clustering demonstrates that the transition clusters express the marker genes of the associated stable clusters and that the marker genes of each transition cluster are expressed in the associated stable clusters (Figure 2F). A similar observation was obtained for the myelopoiesis data (Supplementary Figure 1). While the identification of transition cells by scGeom does not depend on clustering, we used clustering results to interpret the identified transition cells. When a group of transition cells falls within a cluster, it is likely false positive, if the clustering is considered ground truth such as the expert annotated data. On the other hand, if the clustering is due to an arbitrarily initial analysis of the data, such a group of transition cells could help improve the clustering result by separating the cluster into which it falls. Furthermore, we observe a similar correspondence between potential transition cells and negative curvature or high $H_{1}$ total persistence in several other datasets (Supplementary Figures 2 and 3).

### Topological signature reflects differentiation potential

In addition to examining the structures of cell networks, we further explore the relationship between cell states and the structures of gene networks. A prior knowledge-based gene network [13] was assigned to each cell where the node weights were determined by the gene expression levels in the corresponding cell. For each cell, persistent homology was calculated in this node-weighted gene network using vertex-based clique complex filtration where the edge filtration value is determined as the smaller weights of its two nodes.

**Figure 3:**
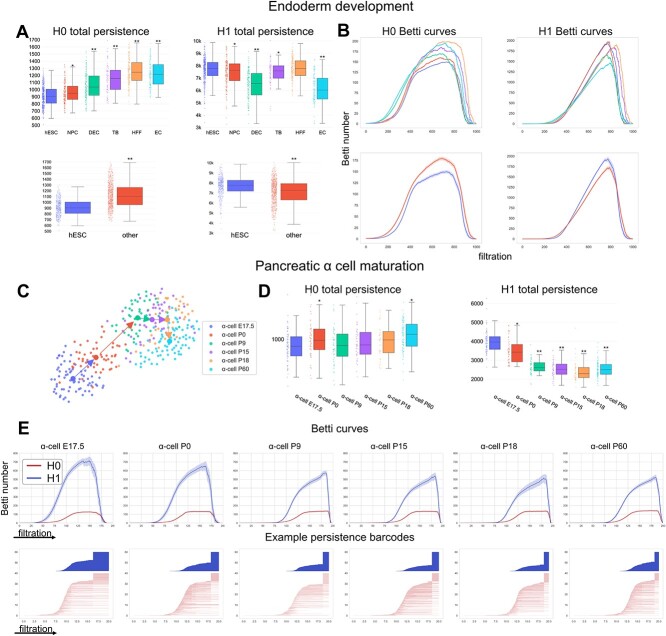
Topological analysis of developmental potential. (**A**) For an scRNA-seq data of human definitive endoderm development, the total persistence of $H_{0}$ and $H_{1}$ persistence barcodes were computed from a vertex-based clique complex of prior knowledge-based gene network (hESC: H1 and H9 human embryonic stem cells, NPC: neuronal progenitor cells, DEC: definitive endoderm cells, TB: trophoblast-like cells, HFF: human foreskin fibroblasts, EC: endothelial cells). * and ** indicate $P$-values less than 0.05 and 1e-10, respectively, for Wilcoxon tests between hESC and other cell types. (**B**) Average $H_{0}$ and $H_{1}$ Betti curves for the detailed cell types and for hESC versus all other cell types. For the latter, the 95% confidence intervals for the mean curve are shown. (**C**) An scRNA-seq data of pancreatic $\alpha $ cell maturation where the arrows show the ground truth developmental trajectory. (**D**) The total persistence of $H_{0}$ and $H_{1}$ persistence barcodes computed from vertex-based clique complex of the prior knowledge-based gene network. * and ** indicate $P$-values less than 0.05 and 1e-10, respectively, for Wilcoxon tests between $\alpha $-cell E17.5 and other cell states. (**E**) Average Betti curves for each cell state with 95% confidence intervals of curve mean, and persistence barcodes of example cells from each state.

We first analyzed an scRNA-seq data of human definitive endoderm development [34]. In this dataset, the human embryonic stem cells (hESC) are pluripotent cells that differentiate into several lineage-specific progenitors. Evaluating the persistent homology of cells in each state, we observed an increase in $H_{0}$ total persistence and a decrease in $H_{1}$ total persistence along the differentiation progress (Figure 3A). The $H_{0}$ and $H_{1}$ persistent homology captures connected components and loop-like structures, which indicates that the gene network of pluripotent cells tends to have less isolated components (shorter $H_{0}$ persistence) and differentiated cells tend to be active in a localized part of the gene network (shorter $H_{1}$ persistence). Comparing hESC cells with all other cells also shows significantly lower $H_{0}$ persistence and higher $H_{1}$ persistence in hESC cells (Figure 3A). The Betti curves summarize the number of connected components ($H_{0}$) and one-dimensional holes or loops ($H_{1}$) at every filtration value, which also show that hESC cells have fewer disconnected parts and larger-scale loops with greater coverage of the gene network(Figure 3B).

We also analyzed an scRNA-seq data of mouse pancreatic $\alpha $ cell maturation tracking along one cell lineage [35]. In this dataset, scRNA-seq experiments were performed for pancreatic $\alpha $ cells at different developmental stages including embryonic data 17.5 (E17.5) and postnatal day P0, P9, P15, P18 and P60 (Figure 3C). During the maturation, we also observed a similar pattern with increasing $H_{0}$ persistence and decreasing $H_{1}$ persistence (Figure 3D). The observation is further confirmed in the Betti curves and persistence barcodes of example cells from each maturation stage (Figure 3E). Together, these examples demonstrate that the topological signatures of gene networks reflect the differentiation potential of cells. A similar trend in $H_{0}$ and $H_{1}$ total persistence of gene networks is observed in several other datasets of developing systems (Supplementary Figure 4).

### Topological machine learning improves cell-type classification

Having shown the utility of topological and geometric structures in single-cell data for analyzing transition cells and developmental potential, here we explore the usage of these structures in the general task of cell-type annotations. We used a mouse brain dataset and a mouse kidney dataset from the cell-type annotation subtask in a benchmarking resource [36] with predefined train/test splits. In this task, a predictive model was trained on annotated data to predict cell types from their gene expression profiles.

We performed two topological characterizations of the gene networks for each cell. First, a cell-specific gene network (CSN) was constructed using a correlation-based approach [11] that results in an edge-weighted gene network for each cell. The CSNs were generated from the processed data using the preprocessing pipeline of SingleCellNet [37] to select the potential marker genes of each cell type. Then, we computed persistent homology using an inverse Vietoris-Rips complex-based filtration, which adds $1$-simplexes and subsequently the higher-dimensional simplices with large edge weights first. Second, we utilized a prior knowledge-based gene network ([13]) on the single-cell datasets without gene filtering. The same gene network structure was assigned to each cell, but with different node weights assigned from gene expression levels. In this case, persistent homology was computed based on a vertex-based clique-complex filtration where $0$-simplices and subsequent higher-dimensional simplices with higher weights are added first. Total persistence and persistence entropy [29] of the resulting persistence diagrams were used as topological features for the cells.

**Figure 4:**
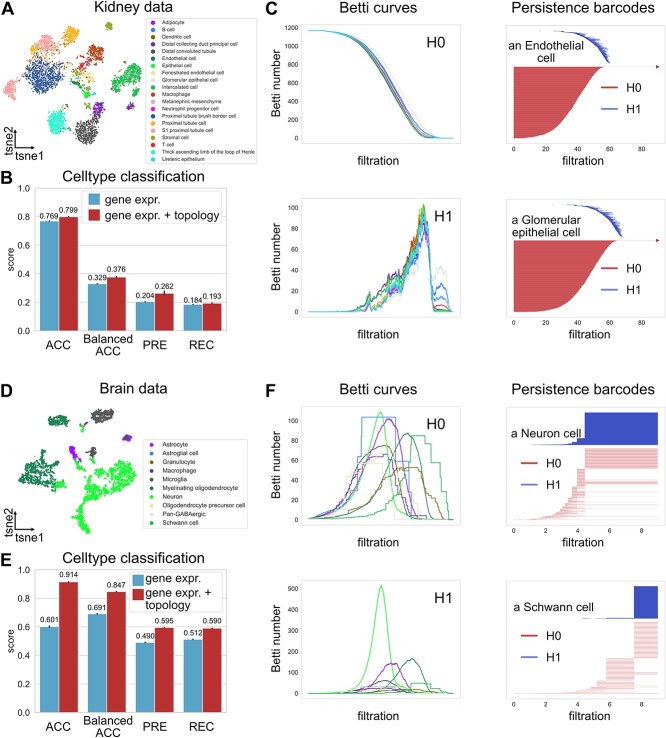
Topology-assisted cell-type annotation (**A**) An scRNA-seq dataset of kidney with annotated cell types. (**B**) The classification performance with or without using topological features in the RF models. The performance is evaluated by accuracy (ACC), adjusted balanced accuracy (Balanced ACC), precision (PRE) and recall (REC) both with macro-average. (**C**) The average $H_{0}$ and $H_{1}$ Betti curves for each cell-type and persistence barcodes of two example cells for the Vietoris-Rips filtration on the cell-specific gene networks. (**D**, **E**) The classification performances on an scRNA-seq dataset of the brain in the RF models. (**F**) The average $H_{0}$ and $H_{1}$ Betti curves for each cell-type and persistence barcodes of two example cells for the vertex-based clique complex filtration on the prior knowledge-based gene network.

In both benchmarks, classification performance is improved with the additional topological features evaluated by accuracy, balanced accuracy, and macro-average precision and recall in both the support vector machine (SVM) and random forest (RF) models (Figure 4 and Table 1). Betti curves and persistence barcodes from several example cells demonstrate the differences in the topological signature of gene networks between different cell types (Figure 4C,F). In the brain dataset, interestingly, we observe significantly longer persistence in the $H_{1}$ persistence barcodes of neuron cells compared to other brain cells, indicating a wider coverage of the gene network and diverse functions. This observation agrees with the diverse signals sent and functions controlled by neuron cells [38]. In these numerical experiments, the inclusion of topological features corrected the predicted types for several cells, especially for neuron cells (Supplementary Figures 5 and 6). Using topological features alone with RF led to inferior performance with adjusted balanced accuracy scores of −0.454 and 0.072 for the kidney and brain datasets, due to the fact that different gene expression profiles could have similar topological structures. Therefore, the structural features could be useful auxiliary features that complement traditional features. Furthermore, we compare with several recent methods for cell-type classification, including deep learning methods scDeepSort [39] and ACTINN [40], a traditional machine learning-based method CellTypist [41], and a method by the DANCE package [36] based on support vector machine (DANCE-SVM). Generally, our results are comparable to other methods with the average accuracy only outperformed by scDeepSort (Table 1). We also investigated the utility of the topological features in unsupervised clustering. If the original topological characteristics are used directly in clustering, due to the lack of the learning process as in the classification task, the improvement is incremental (Supplementary Figure 7). This indicates that some metric learning is needed to effectively use topological features in unsupervised tasks.

**Table 1:** The accuracy of cell-type classification on the mouse kidney and brain datasets

	Kidney dataset				Brain dataset			
Method	Accuracy	Balanced acc.	Precision	Recall	Accuracy	Balanced acc.	Precision	Recall
scDeepSort	0.840(0.004)	0.441(0.007)	0.316(0.051)	0.245(0.027)	0.932(0.024)	0.899(0.014)	0.698(0.118)	0.700(0.108)
CellTypist	0.825(0.033)	0.450(0.065)	0.267(0.064)	0.190(0.040)	0.703(0.009)	0.292(0.014)	0.474(0.039)	0.252(0.019)
ACTINN	0.769(0.029)	0.380(0.044)	0.337(0.025)	0.205(0.014)	0.879(0.087)	0.759(0.061)	0.554(0.056)	0.501(0.054)
DANCE-SVM	0.823(0.000)	0.522(0.000)	0.469(0.000)	0.341(0.000)	0.650(0.000)	0.089(0.000)	0.906(0.000)	0.317(0.000)
SVM (topo)	0.000(0.000)	-0.500(0.000)	0.000(0.000)	0.000(0.000)	0.012(0.000)	-0.270(0.000)	0.252(0.000)	0.024(0.000)
SVM (gene)	0.793(0.000)	0.406(0.000)	0.261(0.000)	0.165(0.000)	0.660(0.000)	0.730(0.000)	0.341(0.000)	0.399(0.000)
SVM (gene+topo)	0.808(0.000)	0.446(0.000)	0.262(0.000)	0.172(0.000)	0.685(0.000)	0.742(0.000)	0.345(0.000)	0.403(0.000)
RF (topo)	0.050(0.010)	-0.454(0.009)	0.051(0.004)	0.011(0.002)	0.645(0.001)	0.072(0.002)	0.422(0.037)	0.186(0.016)
RF (gene)	0.769(0.002)	0.329(0.002)	0.204(0.000)	0.184(0.000)	0.601(0.014)	0.691(0.008)	0.490(0.004)	0.512(0.004)
RF (gene+topo)	0.799(0.003)	0.376(0.013)	0.262(0.055)	0.193(0.006)	0.914(0.006)	0.847(0.004)	0.595(0.003)	0.590(0.002)

Implementations of the package DANCE [36] (version 1.0.0) with default parameters were used to generate the results for scDeepsort, Celltypist, ACTINN and DANCE-SVM where 10 repeated experiments were performed. The average (standard deviation) of accuracy, balanced accuracy, precision and recall across experiments are shown. The last six rows show results using SVM or RF using only topological features (topo), only gene expression levels (gene), or both (gene+topo).

## METHODS

This section describes in detail the methods used. We mainly work on two types of network, a cell network for each scRNA-seq dataset and a cell-specific gene network for each cell in the data. In a cell network, a node represents a cell and the edges indicate similarity in gene expressions. In cell-specific gene networks, a node represents a gene, and the edges denote interactions or correlations between genes. The various $H_{k}$ features refer to the features constructed from the $k$-dimensional persistence barcode.

### Curvature on graphs

Given a metric, the Ricci curvature describes how much the local geometry of a manifold differs from that of ordinary Euclidean space. It measures intrinsic local properties of manifolds such as divergence of geodesics and meeting probabilities of coupled random walks. Several constructions have been introduced to define Ricci curvature on graphs, including ORC [27, 42, 43] and Forman–Ricci curvature. For an edge connecting two nodes, the ORC measures the difference between the edge distance between the nodes and the optimal transport distance between the nodes’ neighborhoods. Let $G=(V,E)$ be an undirected graph with vertices $V=\{v_{i}\}_{i=1}^{n}$ and edges $E=\{e_{ij}\}_{1\leq i,j\leq n}$, a measure is defined for each node describing its local neighborhood such that 


(1) \begin{align*}& m_{v_{i}}(v_{j}; \alpha)=\begin{cases} \alpha,\,\mathrm{if}\, j=i, \\ (1-\alpha)/|N(v_{i})|, \,\mathrm{if}\, i\neq j\in N(v_{i}), \\ 0, \,\mathrm{elsewhere}, \end{cases}\end{align*}

where $N(v_{i})$ is the set of nodes connected to $v_{i}$ in $G$ and $\alpha \in [0,1]$ is the parameter annotating the weight on the center node. The ORC $\kappa _{ij}$ between $v_{i}$ and $v_{j}$ is then defined to be 


(2) \begin{align*}& \kappa^{\mathrm{orc}}_{ij} = 1-\frac{d_{W}(m_{v_{i}},m_{v_{j}})}{d(v_{i},v_{j})},\end{align*}

where $d()$ is the shortest path distance on $G$ and $d_{W}$ is the Wasserstein distance with $d()$ as the ground metric. Specifically, an optimal transport problem is solved: 


(3) \begin{align*}& \begin{aligned} & d_{W}(m_{v_{i}},m_{v_{j}})=\inf\limits_{\pi\in\Pi(m_{v_{i}},m_{v_{j}})}<\pi,C>_{F}, \\ & C_{ij}=d(v_{i},v_{j}), \Pi(a,b)=\{\pi\in\mathbb{R}_{+}^{n\times n}|\pi\mathbf{1}=a,\pi^{\top}\mathbf{1}=b\}. \end{aligned}\end{align*}

### Persistent homology

Persistent homology [19–21] provides a comprehensive multiscale topological characterization by tracking the appearance and disappearance of homology groups through a filtration which is a growing sequence of simplicial complexes defined on the data. An abstract $k$-simplex is a set of $k+1$ vertices denoted $\sigma ^{k}=\{v_{0},\cdots ,v_{k}\}$. Any subset $\sigma ^{\prime}\subseteq \sigma ^{k}$ is called a face of $\sigma ^{k}$. An abstract simplicial complex $K$ is a set of simplices satisfying that all faces of any simplex in $K$ are also in $K$. A filtration of a simplicial complex $K$ is a nested sequence of its subcomplexes: 


(4) \begin{align*}& \emptyset=K^{0}\subset K^{1}\subset\cdots\subset K^{n}=K.\end{align*}

A $k$-chain of a simplicial complex $K$ denoted by $c_{k}$ is a formal sum of $k$-simplices in $K$ with coefficients from a chosen set, for example, $\mathbb{Z}_{2}$. The $k$-chains of $K$ forms a group called the $k$th chain group denoted by $C_{k}(K)$. The $k$-chains are connected by a linear boundary operator: 


(5) \begin{align*}& \begin{aligned} & \partial_{k}: C_{k}(K)\rightarrow C_{k-1}(K), \\ & \partial_{k}(\sigma^{k})=\sum\limits_{i=0}^{k}(-1)^{i}\hat{\sigma}^{k-1}_{i}, \end{aligned}\end{align*}

where $\hat{\sigma }^{k-1}_{i}$ is a face of $\sigma ^{k}$ obtained by removing vertex $i$. Based on the boundary operator, two groups are defined, the kernel of $\partial _{k}$, $Z_{k}(K)=\mathrm{ker}(\partial _{k})$ whose elements are called $k$-cycles and the image of $\partial _{k+1}$ denoted by $B_{k}(K)$ also called the $k$th boundary group. The $k$th homology group is then defined as the quotient group $H_{k}(K)=Z_{k}(K)/B_{k}(K)$ whose rank $\mathrm{rank}(H_{k}(K))=\mathrm{rank}(Z_{k}(K))-\mathrm{rank}(B_{k}(K))$ is also the $k$th Betti number of $K$ representing the number of $k$-dimensional holes in $K$. On the filtration of $K$, the $p$-persistent $k$th homology group is defined as 


(6) \begin{align*}& H_{k}^{p}(K^{i})=Z_{k}(K^{i})/(B_{k}(K^{i+p})\cap Z_{k}(K^{i})),\end{align*}

which intuitively represents a topological feature observed at filtration step $i$ and persists through step $i+p$. An equivalence class in $H_{k}(K^{i})$ that persists through $H_{k}(K^{j})$ but is not present in $H_{k}(K^{i-1})$ or $H_{k}(K^{j+1})$ results in a persistence pair conveniently represented as the interval $[x_{i},x_{j})$ often called the birth-death pairs, where $x_{i}$ and $x_{j}$ are the filtration values of $K^{i}$ and $K^{j}$, respectively. Persistent homology characterization of a dataset results in a collection of such birth-death pairs and is often visualized as persistence barcodes (plotting each pair as a horizontal bar whose two endpoints correspond to the birth and death values) or persistence diagrams (plotting each pair as a point in 2D).

### Data preprocessing and curvature computation

The raw single-cell data were first preprocessed by normalizing total counts in every cell and log1p transformation ($\log (1+x)$). Principal component analysis is then performed with the selected highly variable genes. A $k$-nearest-neighbor graph is constructed based on the Euclidean distance in the space of top principal components. The ORC is computed on this cell network with the $\alpha $ parameter (the mass portion assigned to the center cell when determining a mass distribution representing the neighborhood of the cell) set to 0.5. All preprocessing was performed using the Scanpy package [44].

### Topology of cell networks

To characterize the topological structures of the cell network for each cell, we use two approaches, a local persistent homology and a relative persistent homology. Local persistent homology aims to capture the local structure surrounding a cell in the cell network. Here, we adopt a simple approach by computing the regular persistent homology on a sub-network surrounding a cell defined by either the top $k$ nearest neighbors or a distance cutoff. This approach has been shown to be effective in capturing local topological structures in various applications, such as biomolecular structure analysis [24]. Relative persistent homology [28], on the other hand, captures the significance of a cell or the neighborhood of a cell in assembling the global structure of the dataset. Let $\emptyset =K^{0}\subset K^{1}\subset \cdots \subset K^{n}=K$ be a filtration of the entire dataset. For cell $i$, we define a subcomplex $L_{i}$ that contains only this cell or its local neighborhood on the network. Then, relative persistent homology examines the homology of the relative chain groups, which are quotient groups $C_{k}(K^{j})/C_{k}(K^{j}\cap L_{i})$. The importance of cell $i$ on assembling the global dataset structure is then quantified by computing the Wasserstein distance between the relative persistent homology diagrams and the regular persistent homology diagram of the entire dataset. For both approaches, we used the Vietoris-Rips filtration with the Euclidean distance between cells in their PCA embeddings.

### Topology of gene networks

Here, we consider two types of gene networks, a cell-specific gene network in the form of edge-weighted networks and a prior knowledge-based gene network in the form of node-weighted networks.

The CSN package [11] is used to construct a cell-specific gene network for each cell on the top highly variable genes in the dataset. The core statistic in this method evaluates the local association between each pair of genes for each cell. Specifically, for cell $k$ and genes $x$ and $y$, 


(7) \begin{align*}& \rho_{xy}^{(k)}=n_{xy}^{(k)}/n-(n_{x}^{(k)}/n)(n_{y}^{(k)}/n),\end{align*}

where $n$ is the total number of cells. The parameter $n_{x}^{(k)}$ is predefined and induces an interval $I_{x}^{(k)}$ of the expression of gene $x$ in the top $n_{x}^{(k)}$ cells whose gene $x$ expression is the closest to cell $k$. Similarly, an interval for gene $y$, $I_{y}^{(k)}$ is determined by $n_{y}^{(k)}$. Then $n_{x}y^{(k)}$ counts the number of cells in whose expressions of gene $x$ and gene $y$ both fall in the two intervals, respectively. Here, we used the top 1000 highly variable genes and used the default parameter values in the CSN method [11] with $n_{x}^{(k)}=n_{y}^{(k)}=0.1n$ and the significant level set to $0.01$. The cell-specific gene networks constructed are edge-weighted networks. Denoting the network of a cell by $G=(V,E,W^{(e)})$, we compute persistent homology with the Vietoris-Rips filtration 


(8) \begin{align*}& VR(\delta)=\{\sigma: \forall \sigma^{(1)}=(i,j)\subseteq\sigma, W^{(e)}_{ij}\geq\delta \,\,\mathrm{and}\,\, (i,j)\in E\}.\end{align*}

The filtration is computed from $\delta =\delta _{max}=\max \{W^{(e)}\}$ to $\delta =0.$ The resulting persistence pairs $[b_{i},d_{i})_{i}$ are transformed to $[\delta _{max}-b_{i},\delta _{max}-d_{i})_{i}$.

For the prior knowledge-based gene network, a base network is first assigned to each cell, and the gene expression levels in each cell are assigned as node weights. Denoting the network of a cell by $G=\{V,E,W^{(v)}\}$, persistent homology is computed using the vertex-based clique complex filtration 


(9) \begin{align*}& \begin{aligned} CL&(\delta)=\{\sigma: \forall \sigma^{(0)}_{i}\subseteq \sigma, W^{(v)}_{i}\geq\delta ; \forall \sigma^{(1)}=(i,j)\subseteq\sigma, \\ &\min\{W^{(v)}_{i}, W^{(v)}_{j}\}\geq\delta \,\,\mathrm{and}\,\,(i,j)\in E\}. \end{aligned}\end{align*}

Similar to the edge-weighted network, the filtration is computed from $\delta =\delta _{max}=\max \{W^{(v)}\}$ to $\delta =0$. The resulting persistence pairs $[b_{i},d_{i})_{i}$ are also transformed into $[\delta _{max}-b_{i},\delta _{max}-d_{i})_{i}$. The log1p transformed gene expression levels are used to assign node weight and the full dataset without gene filtering is used with the knowledge-based gene network.

The calculation of filtration and persistent homology was based on the packages Gudhi [45], Dionysus2 [46] and Ripser [47].

### Featurization of persistence diagrams

For dimension $k$, persistent homology computation results in a collection of $n^{(k)}$ persistence pairs $[b^{(k)}_{i},d^{(k)}_{i})_{i=1}^{n^{(k)}}$ where $b^{(k)}_{i}$ and $d^{(k)}_{i}$ are the filtration values corresponding to the birth and death of a topological feature ($k$-dimensional holes). Several summaries and features are derived from the persistence pairs. Total persistence describes the overall significance of topological features in the data and is computed as 


(10) \begin{align*}& L^{(k)}=\sum_{i=1}^{n} (\bar{d}^{(k)}_{i}-b^{(k)}_{i}), \bar{d}^{(k)}_{i}=\min\{d^{(k)}_{i}, \delta_{max}\}.\end{align*}

The persistence entropy [29, 30] describes the heterogeneity of persistence similar to the Shannon entropy and is stable with respect to small perturbations in the input space. It has been applied successfully to molecular sciences [31]. Specifically, it is computed as 


(11) \begin{align*}& E^{(k)}=-\sum_{i=1}^{n^{(k)}}\frac{l^{(k)}_{i}}{L^{(k)}}\log_{2}\left(\frac{l^{(k)}_{i}}{L^{(k)}}\right),\end{align*}

where $l^{(k)}_{i}=\bar{d}^{(k)}_{i}-b^{(k)}_{i}$ is the persistence of the $i$th pair. In addition to global summaries, Betti curves describe structural changes along the filtration and are convenient for illustrating the average behavior of a group of persistence barcodes. For a filtration value $\delta $, the $k$-dimensional Betti curve is computed as 


(12) \begin{align*}& BC^{(k)}(\delta)=\#\{[b^{(k)}_{i},d^{(k)}_{i}):\delta\in[b^{(k)}_{i},d^{(k)}_{i})\}.\end{align*}

Betti curve is computed on a discretization of the filtration interval $[0,\delta _{max}]$. The Gudhi package [45] was used to calculate persistence entropy and Betti curves.

### Machine learning and evaluation metrics

In the application of cell-type classification, the implementations of the RF model (RandomForestClassifier) and the SVM in the scikit-learn package [48] were used. In both models, the *class_weight* parameter was set to *balanced*. The number of trees parameter *n_estimators* was set to 5000 in the RF and the regularization parameter *C* was set to 0.5 in the SVM. All other parameters are set to default values. The classification results on the test set were evaluated using commonly used metrics, including accuracy: 


(13) \begin{align*}& (1/N)\sum_{i}1(\hat{y}_{i}=y_{i});\end{align*}

balanced accuracy (adjusted): 


(14) \begin{align*}& \left(1/\sum_{i}\frac{1}{N_{c(i)}}\right)\sum_{i}1(\hat{y}_{i}=y_{i})\frac{1}{N_{c(i)}}-\frac{1}{C};\end{align*}

precision (macro-average): 


(15) \begin{align*}& \frac{1}{C}\sum_{c=1}^{C}\frac{|\hat{Y}_{c}\cap Y_{c}|}{|\hat{Y}_{c}|};\end{align*}

and recall (macro-average): 


(16) \begin{align*}& \frac{1}{C}\sum_{c=1}^{C}\frac{|\hat{Y}_{c}\cap Y_{c}|}{|Y_{c}|}.\end{align*}

Here, $N$ is the total number of samples, $N_{c(i)}$ is the number of samples of the same class as sample $i$ in the ground truth, $y_{i}$ is the true label of sample $i$, $\hat{y}_{i}$ is the predicted label of sample $i$, $C$ is the number of classes, $Y_{c}$ is the set of samples of class $c$ in the ground truth, $\hat{Y}_{c}$ is the set of sample predicted to be class $c$ and $1()$ is the indicator function.

## CONCLUSION AND DISCUSSION

To exploit the complex underlying structures in scRNA-seq data, we developed scGeom, a tool to derive topological and geometric signatures from cell networks and the gene networks associated with each cell. It utilizes ORC, local persistent homology, relative persistent homology, and persistent homology filtrations for edge-weighted and node-weighted networks. The utility of these structural characterizations has been demonstrated on real scRNA-seq datasets for identifying transition cells, quantifying pluripotency or differentiation potential of cells, and assisting in the classification of cells.

Persistent homology is used as a structural descriptor in this work without tracing back to individual cells or genes from topological signatures. Recently, several methods have been proposed to connect the topological features with the input data [49–51] which could help to interpret the topological features in terms of cells or genes. The topological structures are compared as topological summaries between large-scale gene networks in this work. When comparing small-scale networks in the future, such as specific pathways, two networks could have the same structure but different arrangements of genes, which will result in identical topological structures. This could be addressed by a recent work that compares topological summaries while considering differences in the original data [52].

This work presents one of the early efforts to apply persistent homology to scRNA-seq data. The methods are also potentially applicable to other single-cell omics data [53], given some similarity measurement between cells or association scores between features. With recent developments in multiparameter persistent homology [54], different metrics can be considered simultaneously in complex data such as single-cell multi-omics data with multiple similarity measurements and spatial transcriptomics data with spatial distance and gene expression similarities.

For the curvature and topology of cell networks, while we use the commonly used combination of PCA and Euclidean distance to characterize the dissimilarity among cells in this work, there are other options, such as cosine dissimilarity. With different combinations of the number of principal components, number of neighbors, and different dissimilarity measurements, including Euclidean distance, cosine dissimilarity and Pearson’s correlation coefficient, generally stable results are obtained (Supplementary Figures 8–10). For the construction of gene networks, here we used a prior knowledge-based network [14] and a cell-specific gene network [11] for their efficiency and comprehensive coverage of genes. Alternatively, the gene regulatory networks specifically inferred for each scRNA-seq dataset could also be used [55]. For unsupervised tasks such as clustering, metric learning could help to effectively use the topological features of gene networks and can be potentially integrated into existing clustering methods [56–59]. For characterizing the cell networks, both curvature and local persistent homology are computed on neighborhood of each cell with fixed size, and therefore scales linearly to cell numbers. The relative persistent homology could become computationally costly with increasing cell number due to its characterization of global structure relative to each cell neighborhood. For the cell-specific gene network, the computational cost also scales linearly to the cell number due to the fixed size of the gene networks.

**Key Points**

- We developed scGeom, a computational tool for analyzing the underlying structures in scRNA-seq data based on the emerging topological and geometric data analysis methods.
- The method relates structures in the data to functions of cells that connect topological and geometric features of cell or gene networks to reveal transition cells and differentiation potential.
- The features resulting from the topological and geometric characterizations can serve as additional cellular descriptors. The addition of such features has improved performance in cell-type classification due to the additional higher-order information captured.

## AUTHOR CONTRIBUTIONS

ZC conceived the method. TH and ZC implemented the methods, performed the analysis, interpreted the results and wrote the manuscript.

## FUNDING

This work is supported by a National Science Foundation grant DMS-2151934 and a startup grant from NC State University. TH is partly supported by the Center for Research in Scientific Computation at NC State University.

## DATA AND CODE AVAILABILITY

All datasets used are publicly available and are summarized in Table 2. The Dance (v1.0.0) implementations of scDeepSort, CellTypist, ACTINN and its internal classifier DANCE-SVM with default parameters were used to generate the comparison results. The package scGeom is available at https://github.com/zcang/scGeom.

**Table 2:** A summary of the datasets used in this paper

Data	Cell#	Access	Presented	Features	Seq. tech.
Myelopoiesis [32]	375	GSE70245	Figure 2(A,B)	top PCs	Fluidigm C1
iPSC [33]	1081	Suppl. of [33]	Figure 2(C–F)	top PCs	RT-PCR
Endoderm [34]	1018	GSE75748	Figure 3(A,B)	all genes	Fluidigm C1
Pancreatic-$\alpha $ [35]	322	GSE87375	Figure 3(C–E)	all genes	Smart-seq2
Pancreatic-$\beta $ [35]	562	GSE87375	Supplementary Figure 4(C)	all genes	Smart-seq2
Kidney (annot.) [60]	4885	Dance [36]	Figure 4(A–C)	all genes	Microwell-Seq
Brain (annot.) [60]	3448	Dance [36]	Figure 4(D–F)	all genes	Microwell-Seq
Planaria 1 [61]	5354	GSE103633	Supplementary Figure 2(A)	top PCs	Drop-seq
Planaria 2 [61]	13 281	GSE103633	Supplementary Figure 2(B)	top PCs	Drop-seq
Planaria 3 [61]	4341	GSE103633	Supplementary Figure 3(C)	top PCs	Drop-seq
Planaria 4 [61]	11 319	GSE103633	Supplementary Figure 3(D)	top PCs	Drop-seq
Bone marrow [60]	3105	GSE108097	Supplementary Figure 2(C)	top PCs	Microwell-Seq
Dentate gyrus [62]	3583	GSE95315	Supplementary Figure 2(D)	top PCs	10x Chromium
Kidney [63]	1533	GSE107585	Supplementary Figure 3(A)	top PCs	10x Chromium
Placenta [60]	1001	GSE108097	Supplementary Figure 3(B)	top PCs	Microwell-Seq
Fibroblast [64]	355	GSE67310	Supplementary Figure 4(A)	all genes	Fluidigm C1
Oligo. diff. [65]	4930	GSE75330	Supplementary Figure 4(B)	all genes	Fluidigm C1

## Supplementary Material

Supplementary_Figure_bbae176

## Author Biographies

**Tram Huynh** is an undergraduate student in Statistics and Applied Mathematics at North Carolina State University. Her current research interests are statistical mathematics and data analysis.

**Zixuan Cang** is an assistant professor at Department of Mathematics, North Carolina State University.
