# Supplementary material for: Topological and geometric analysis of cell states in single-cell transcriptomic data
Source: Brief Bioinform. 2024 Apr 18;25(3):bbae176. doi: 10.1093/bib/bbae176 (PMC11024518; doi:10.1093/bib/bbae176)
Supplement: Supplementary_Figure_bbae176 [file supplementary_figure_bbae176.pdf]

Supplementary Figures

Topological and geometric analysis of cell states in single-cell transcriptomic data
Tram Huynh and Zixuan Cang

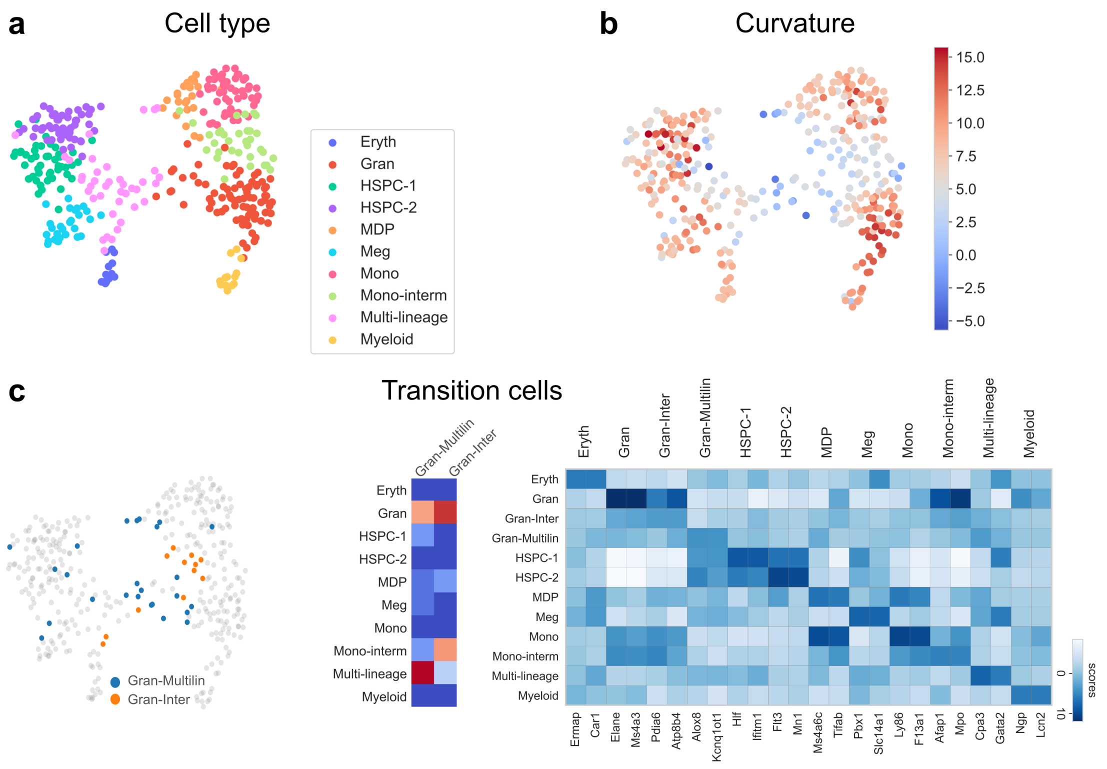

SFigure 1. Marker gene analysis of the myelopoiesis dataset. **a** The predetermined cell types. **b** The curvature computed on the cell network. **c** Transition cells are determined by the curvature values. The identified transition cells are then clustered into two classes by unsupervised clustering. The top two genes of each class is shown in the heatmap where the each class from the original cell type assignment contains the original cells excluding the identified transition cells.

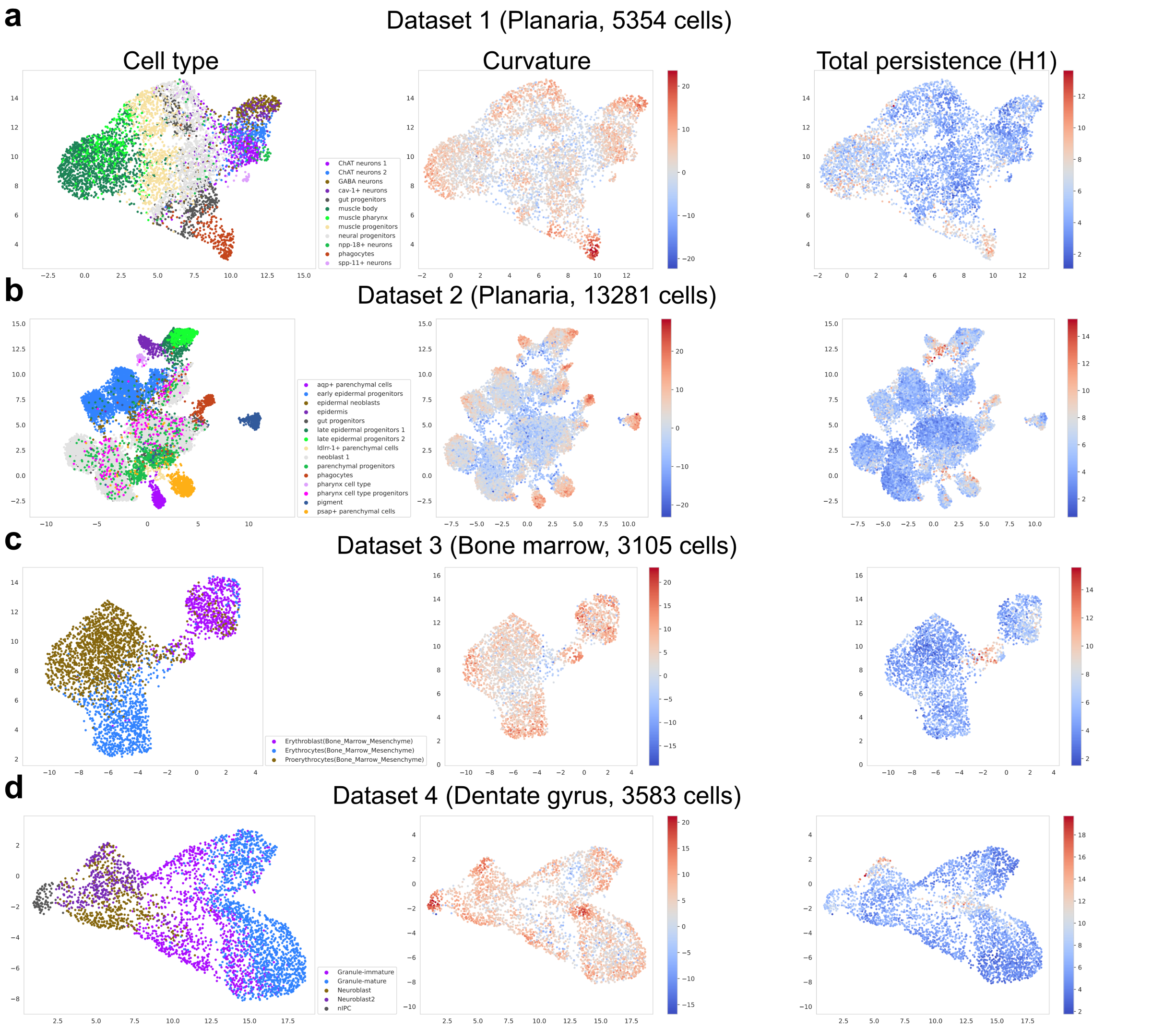

SFigure 2. Examples of cell network curvature and total persistence indicating transition cells. Note the negative curvature and high persistence that highlight the cells between known cell types. Datasets 1 and 2 are from "Plass, Mireya, et al. "Cell type atlas and lineage tree of a whole complex animal by single-cell transcriptomics." Science 360.6391 (2018): eaaq1723." with accession number GSE103633. Dataset 3 is from "Han, Xiaoping, et al. "Mapping the mouse cell atlas by microwell-seq." Cell 172.5 (2018): 1091-1107." with accession number GSE108097. Dataset 4 is from "Hochgerner, Hannah, et al. "Conserved properties of dentate gyrus neurogenesis across postnatal development revealed by single-cell RNA sequencing." Nature neuroscience 21.2 (2018): 290-299." with accession number GSE95315.

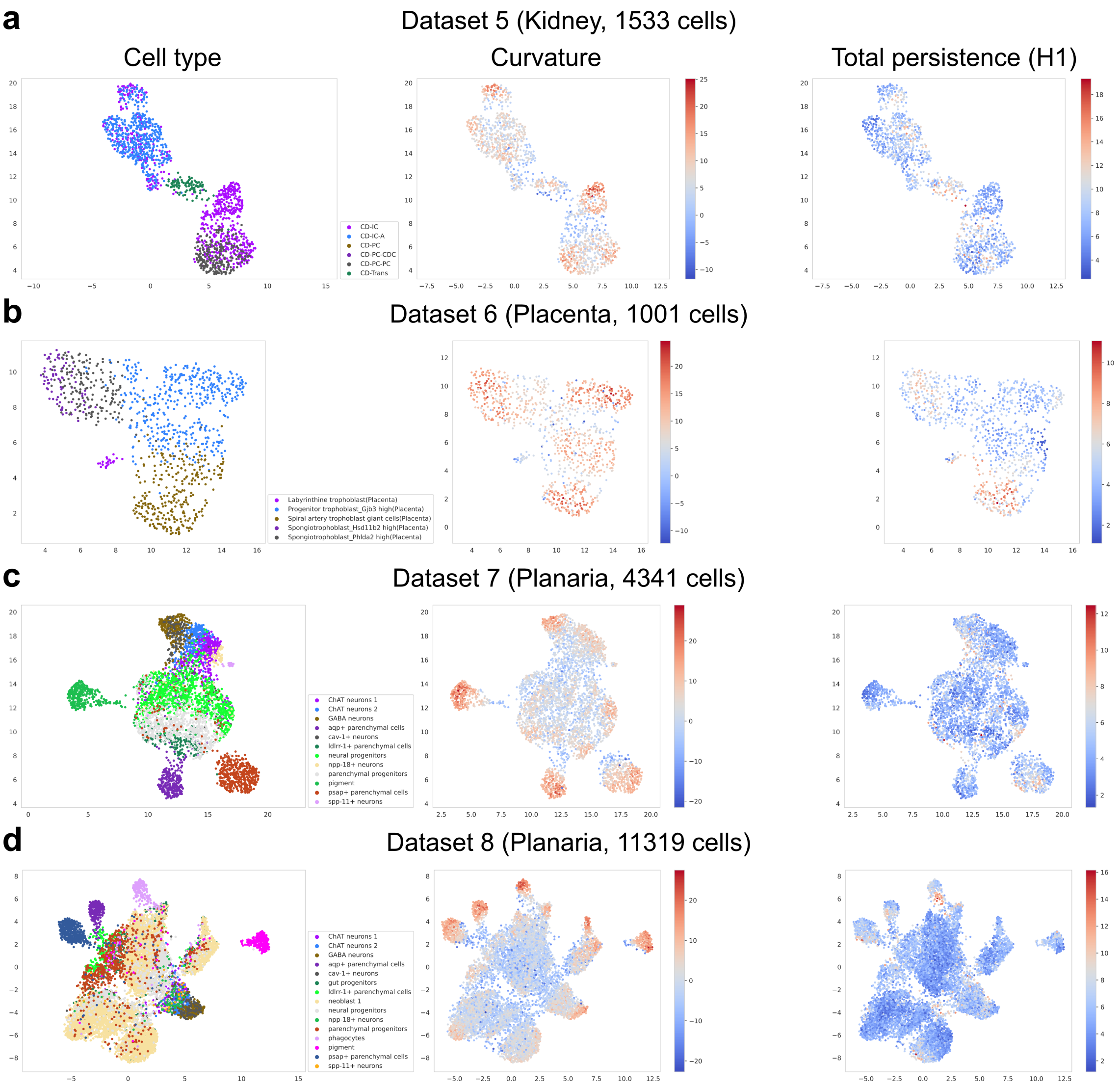

SFigure 3. Examples of cell network curvature and total persistence indicating transition cells. Note the negative curvature and high persistence that highlight the cells between known cell types. Dataset 5 is from "Park, Jihwan, et al. "Single-cell transcriptomics of the mouse kidney reveals potential cellular targets of kidney disease." Science 360.6390 (2018): 758-763." with accession number GSE107585. Dataset 6 is from "Han, Xiaoping, et al. "Mapping the mouse cell atlas by microwell-seq." Cell 172.5 (2018): 1091-1107." with accession number GSE108097. Datasets 7 and 8 are from "Plass, Mireya, et al. "Cell type atlas and lineage tree of a whole complex animal by single-cell transcriptomics." Science 360.6391 (2018): eaaq1723." with accession number GSE103633.

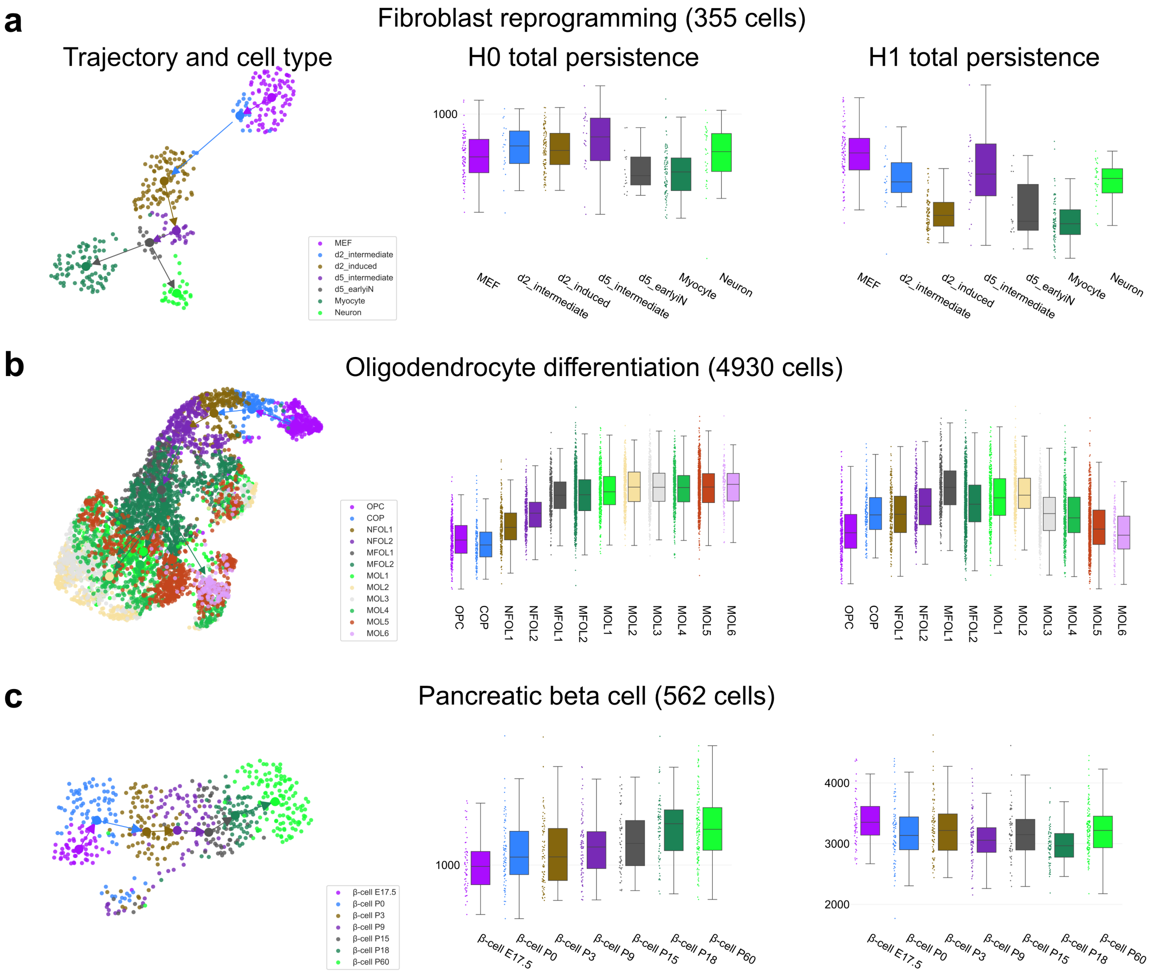

SFigure 4. Examples of cell specific gene network topological features and their relationship with the developmental potentials. The fibroblast reprogramming dataset is from "Treutlein, Barbara, et al. "Dissecting direct reprogramming from fibroblast to neuron using single-cell RNA-seq." Nature 534.7607 (2016): 391-395." with accession number GSE67310. The Oligodendrocyte differentiation dataset is from "Marques, Sueli, et al. "Oligodendrocyte heterogeneity in the mouse juvenile and adult central nervous system." Science 352.6291 (2016): 1326-1329." with accession number GSE75330. The Pancreatic beta cell dataset is from "Qiu, Wei-Lin, et al. "Deciphering pancreatic islet β cell and α cell maturation pathways and characteristic features at the single-cell level." Cell metabolism 25.5 (2017): 1194-1205." with accession number GSE87375.

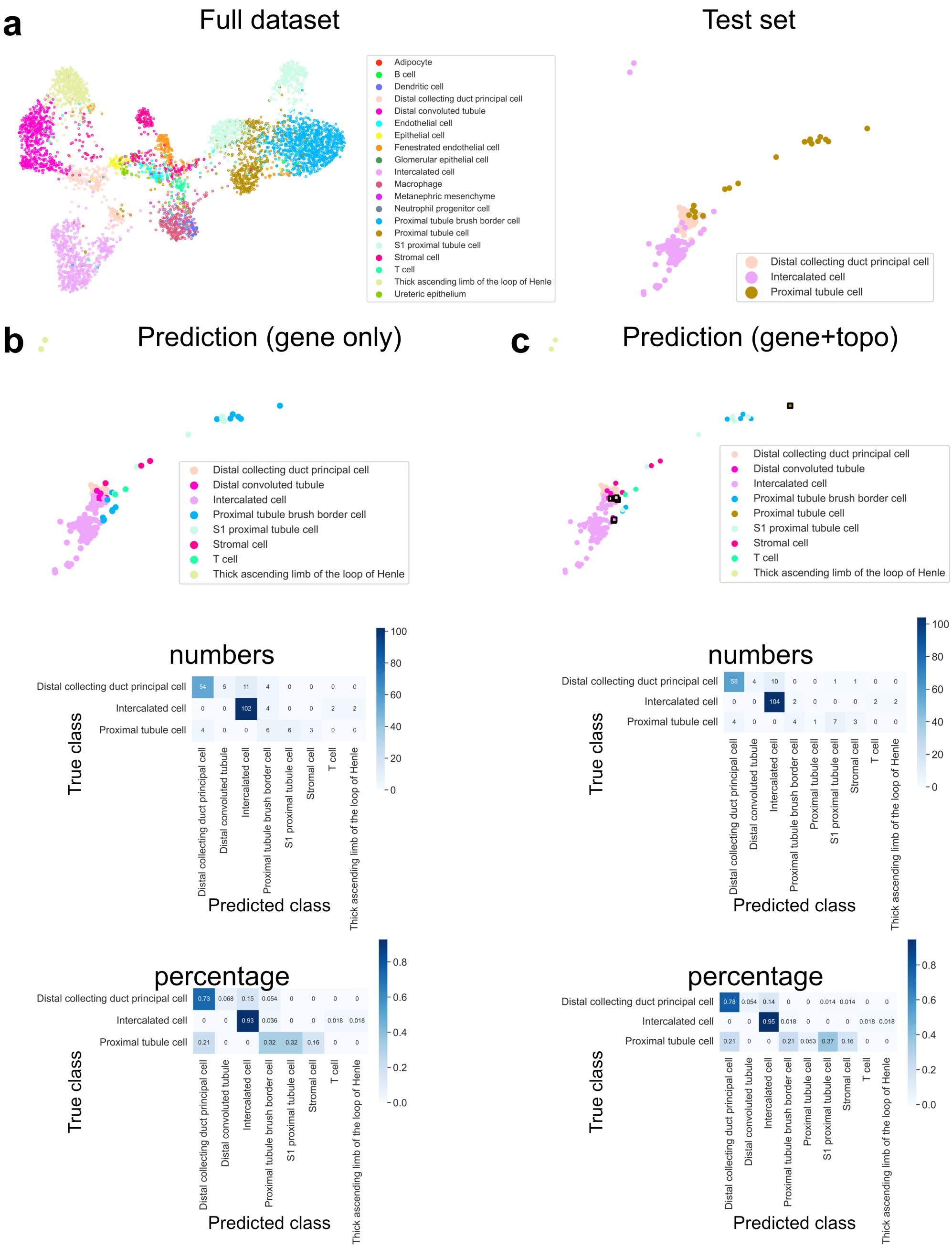

SFigure 5. Classification details of the kidney dataset. **a** The full dataset containing both the training and testing set represented in UMAP embedding, and the test set. **b,c** The prediction results from the two approaches using only gene expression features (b) or using both gene expression features and the topological features (c). The cells plotted as squares are the ones that are only correctly predicted in the corresponding approach. The numbers and percentages of cells in each true class predicted to be each predicted class are shown as heatmaps.

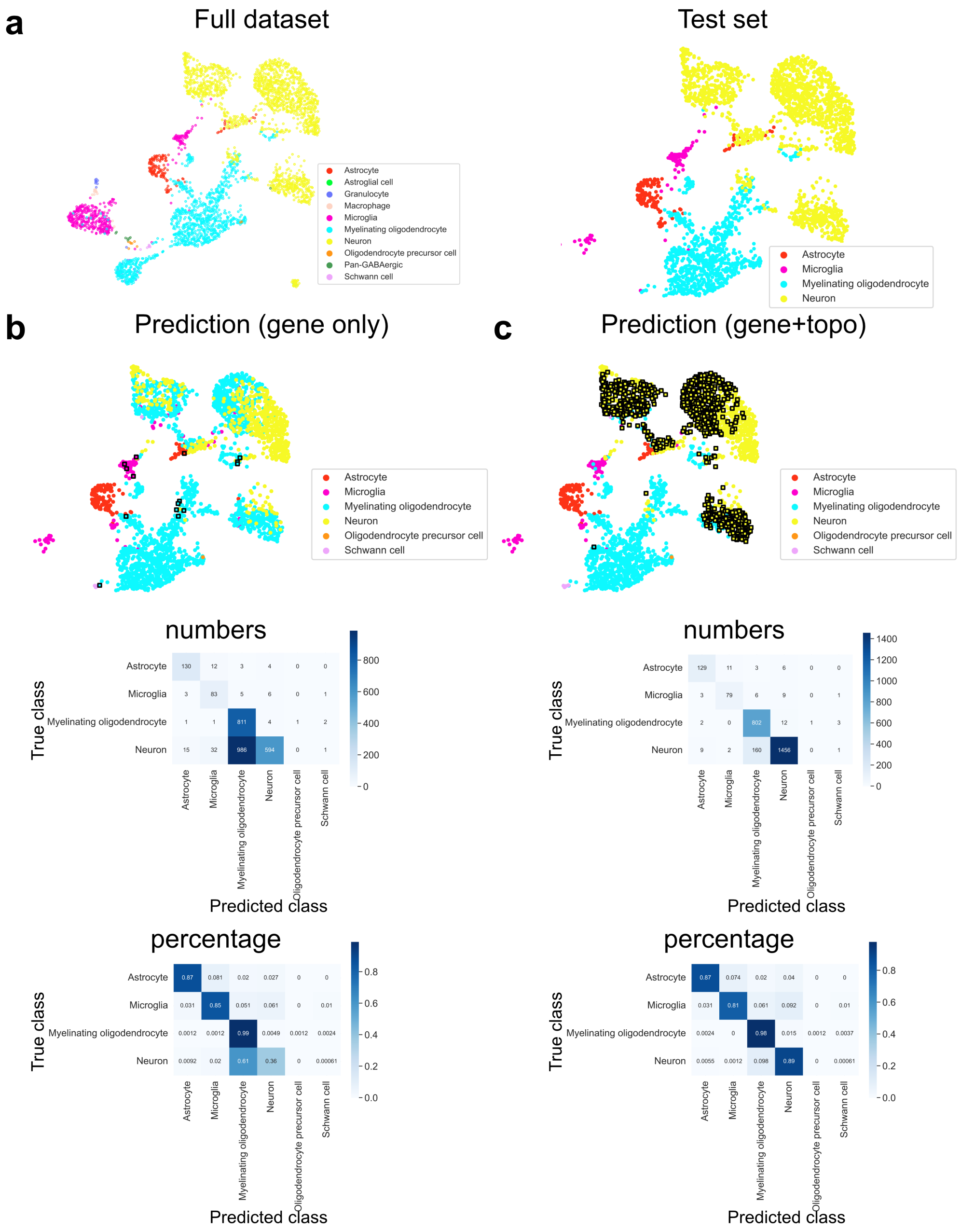

SFigure 6. Classification details of the brain dataset. **a** The full dataset containing both the training and testing set represented in UMAP embedding, and the test set. **b,c** The prediction results from the two approaches using only gene expression features (b) or using both gene expression features and the topological features (c). The cells plotted as squares are the ones that are only correctly predicted in the corresponding approach. The numbers and percentages of cells in each true class predicted to be each predicted class are shown as heatmaps.

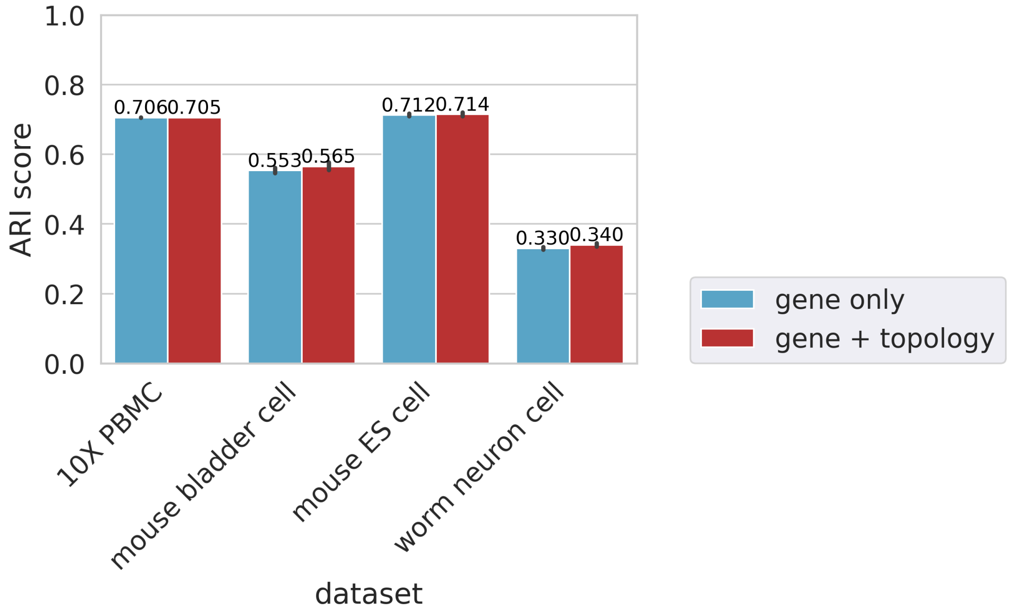

SFigure 7. Clustering performances. The four benchmark datasets are taken from "Ding, Jiayuan, et al. "DANCE: A Deep Learning Library and Benchmark Platform for Single-Cell Analysis." bioRxiv (2022): 2022-10. ". For all datasets, the original data was first normalized and log1p transformed with top 1000 variable genes selected using the Scanpy package. Edge-weighted persistent homology was computed on the gene networks constructed using the package CSN. The total persistence and persistence entropy of H_0 and H_1 barcodes were used as the topological features. In all experiments, for "gene only", the top 30 principal components were used as features, and for "gene + topology", the top 30 PCs and the 4 additional topological features were used as features. Using Scanpy, leiden clustering was performed with resolution=0.5 based on neighbor graphs created with n_neighbors=30. All experiments were repeated 50 times due to the randomness in leiden algorithm with the mean Adjusted Rand Indexes reported.

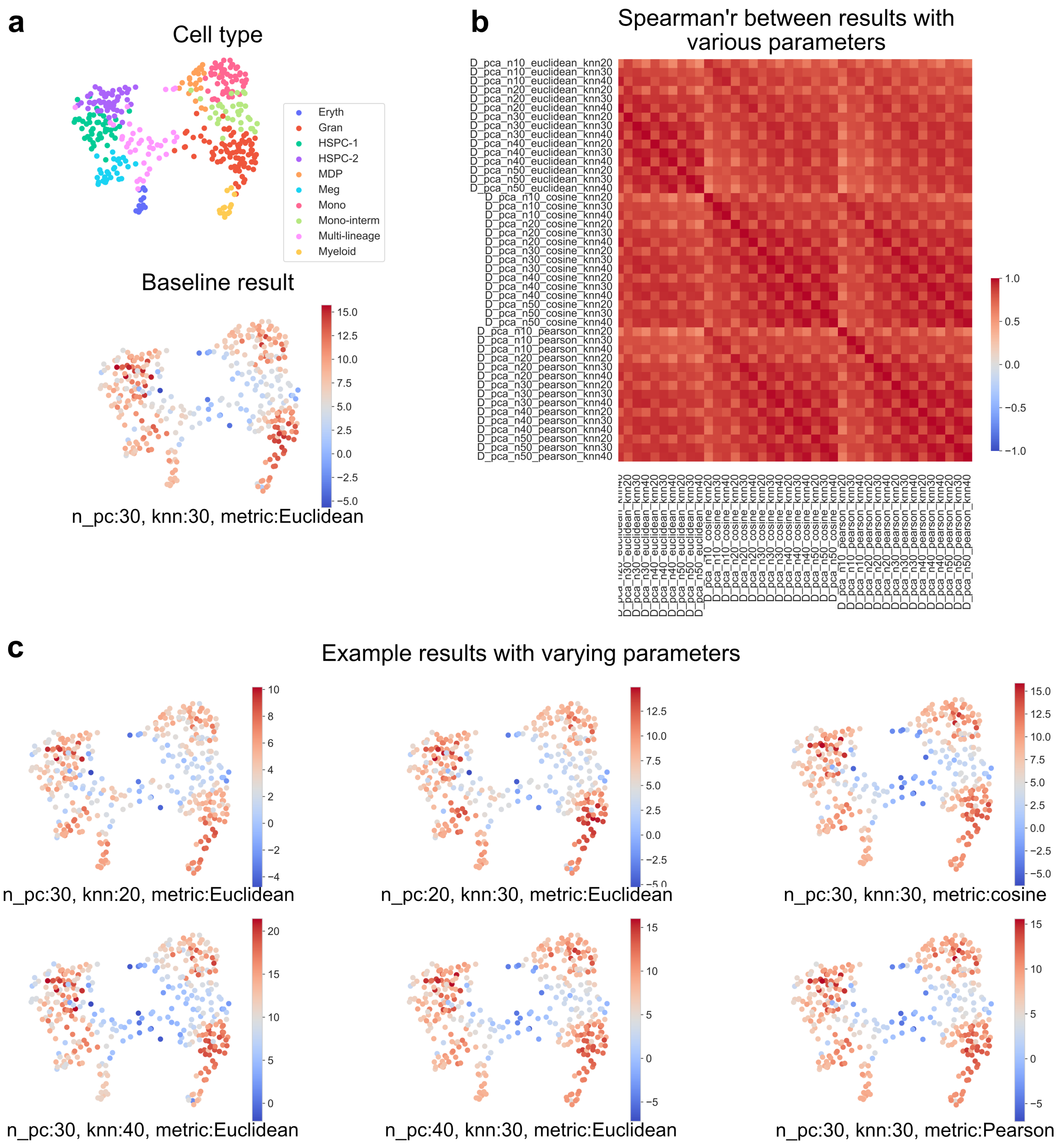

SFigure 8. Curvature obtained from different numbers of principal components, nearest neighbors in knn graph, and metrics used. **a** The cell type and baseline result obtained with 30 pcs, 30 neighbors and Euclidean distance. **b** The Spearman's correlation coefficient among results using various combinations of parameters. **c** Some example results with paramters different from the baseline result.

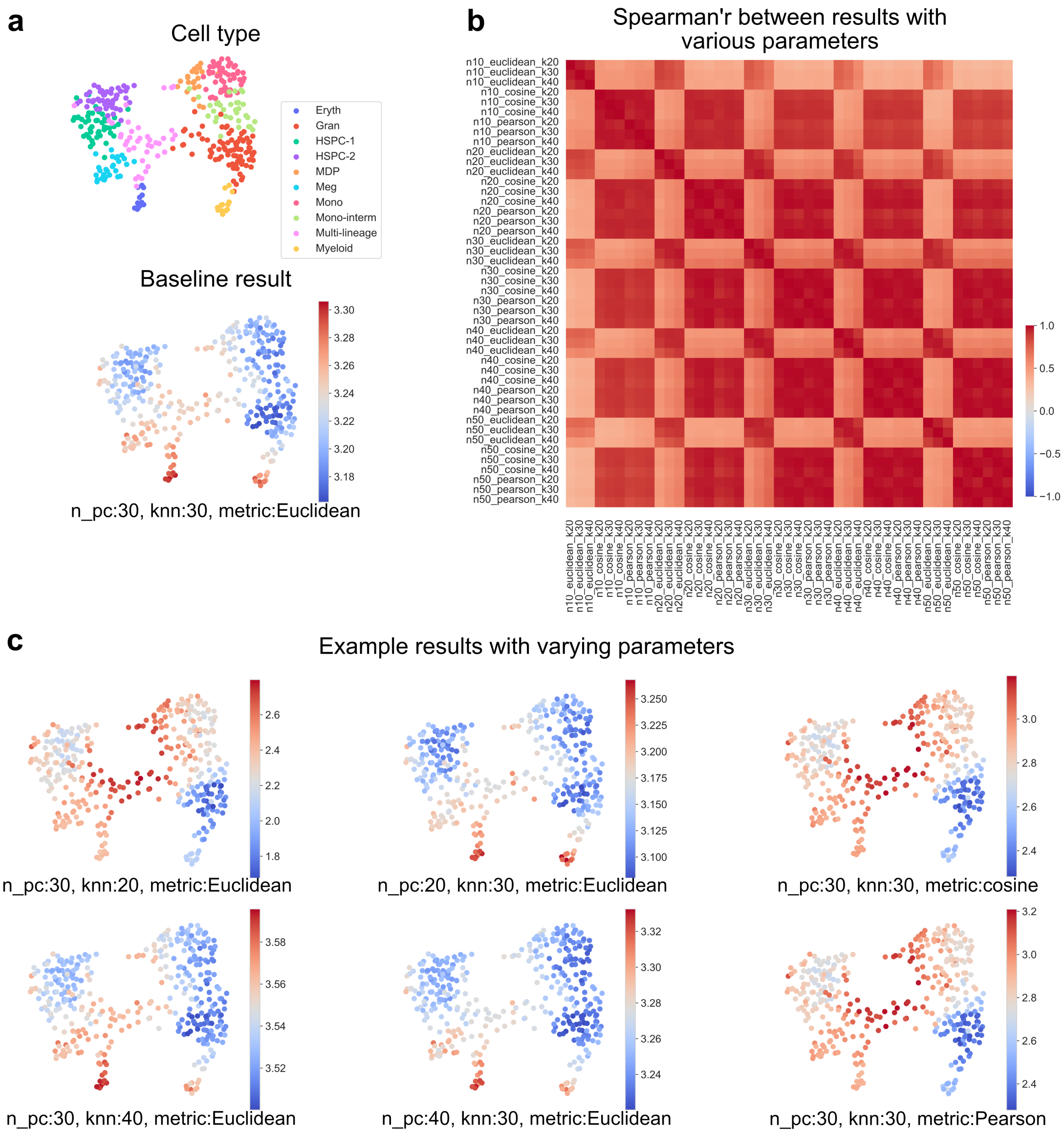

SFigure 9. The H_0 persistence entropy obtained from different numbers of principal components, nearest neighbors in knn graph, and metrics used. **a** The cell type and baseline result obtained with 30 pcs, 30 neighbors and Euclidean distance. **b** The Spearman's correlation coefficient among results using various combinations of parameters. **c** Some example results with paramters different from the baseline result.

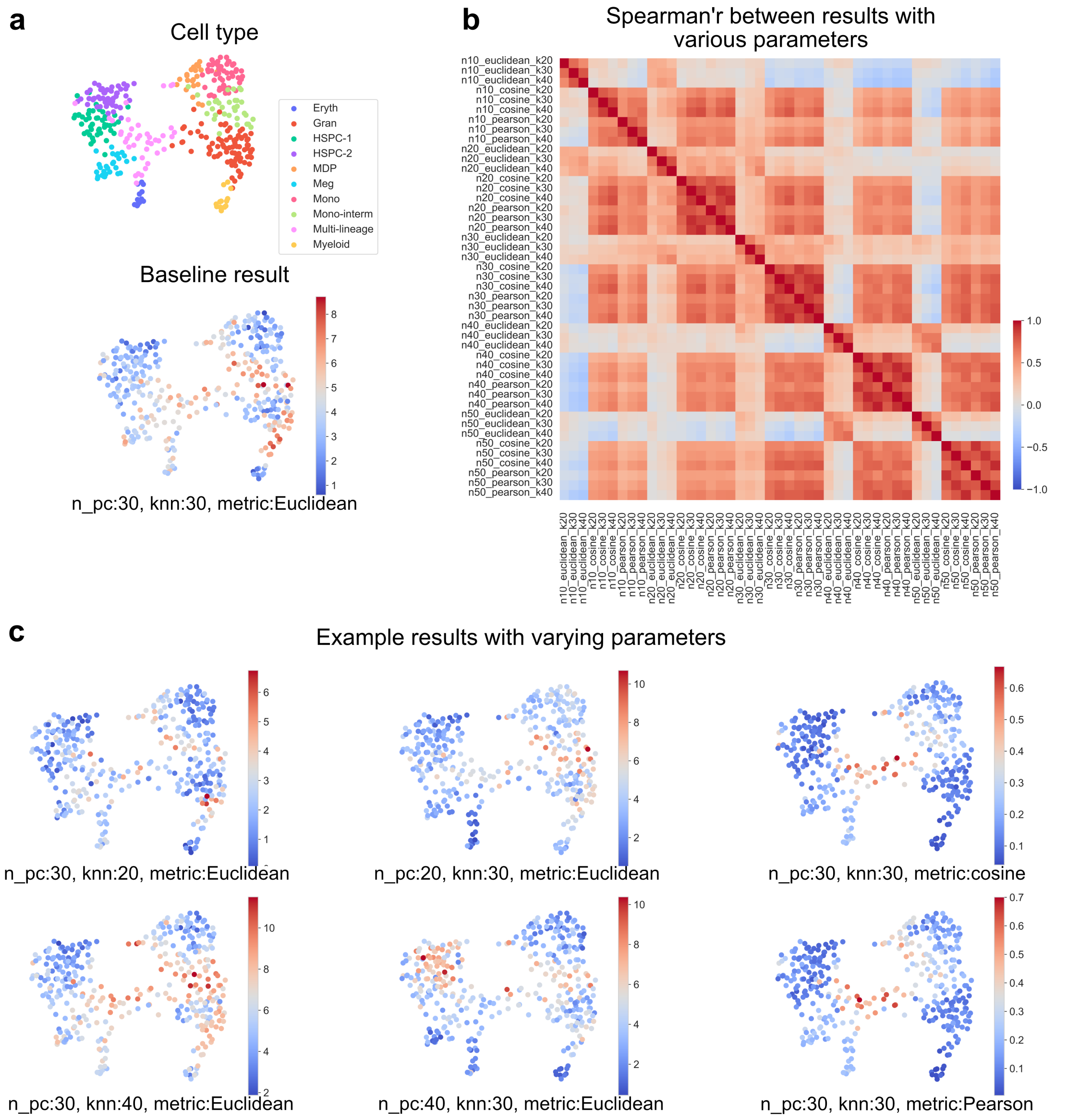

SFigure 10. The H_1 total persistence obtained from different numbers of principal components, nearest neighbors in knn graph, and metrics used. **a** The cell type and baseline result obtained with 30 pcs, 30 neighbors and Euclidean distance. **b** The Spearman's correlation coefficient among results using various combinations of parameters. **c** Some example results with paramters different from the baseline result.
